# Impact of breast cancer molecular subtypes on the incidence, kinetics and prognosis of central nervous system metastases in a large multicentre real-life cohort

**DOI:** 10.1038/s41416-019-0619-y

**Published:** 2019-11-13

**Authors:** Amélie Darlix, Guillaume Louvel, Julien Fraisse, William Jacot, Etienne Brain, Marc Debled, Marie Ange Mouret-Reynier, Anthony Goncalves, Florence Dalenc, Suzette Delaloge, Mario Campone, Paule Augereau, Jean Marc Ferrero, Christelle Levy, Jean-David Fumet, Isabelle Lecouillard, Paul Cottu, Thierry Petit, Lionel Uwer, Christelle Jouannaud, Marianne Leheurteur, Véronique Dieras, Mathieu Robain, Michaël Chevrot, David Pasquier, Thomas Bachelot

**Affiliations:** 1 Department of Medical Oncology, Institut du Cancer de Montpellier (ICM), University of Montpellier, 208 Rue des Apothicaires, 34298 Montpellier, France; 2 Department of Radiation Therapy, Gustave Roussy, 114 Rue Edouard Vaillant, 94800 Villejuif, France; 3 Biometrics Unit, Institut du Cancer de Montpellier (ICM), University of Montpellier, 208 Rue des Apothicaires, 34298 Montpellier, France; 4 Institut de Recherche en Cancérologie de Montpellier (IRCM), INSERM U1194, Institut du Cancer de Montpellier, University of Montpellier 208 Rue des Apothicaires, 34298 Montpellier, France; 5 Department of Medical Oncology, Institut Curie, 26 Rue d’Ulm, 75005 Paris & Saint-Cloud, France; 6 Department of Medical Oncology, Institut Bergonié, 229 Cours de l’Argonne, 33000 Bordeaux, France; 7 Department of Medical Oncology, Centre Jean Perrin, 58 Rue Montalembert, 63011 Clermont Ferrand, France; 8 Department of Medical Oncology, Institut Paoli-Calmettes, 232 Boulevard de Sainte-Marguerite, 13009 Marseille, France; 9 Department of Medical Oncology, Institut Claudius Regaud – IUCT Oncopole, 1 Avenue Irène-Joliot-Curie, 31059 Toulouse, France; 10 Department of Cancer Medicine, Gustave Roussy, 114 Rue Edouard-Vaillant, 94800 Villejuif, France; 11 Department of Medical Oncology, Institut de Cancérologie de l’Ouest Centre René Gauducheau, Boulevard Jacques Monod, 44805 Saint Herblain, France; 12 Department of Medical Oncology, Institut de Cancérologie de l’Ouest, 15 rue André Boquel, 49055 Angers, France; 13 Department of Medical Oncology, Centre Antoine Lacassagne, 33 Avenue de valambrose, 06189 Nice, France; 14 Department of Medical Oncology, Centre François Baclesse, 3 Avenue du Général Harris, 14000 Caen, France; 15 Department of Medical Oncology, Centre Georges François Leclerc, 1 rue Professeur Marion, 21079 Dijon, France; 16 Department of Radiation Oncology, Centre Eugène Marquis, Avenue de la Bataille Flandres-Dunkerque, 35000 Rennes, France; 17 Department of Medical Oncology, Centre Paul Strauss, 3 Rue de la Porte de l’Hôpital, 67000 Strasbourg, France; 18 Medical Oncology Department, Institut de Cancérologie de Lorraine, 6 Avenue de Bourgogne, 54519 Vandœuvre-lès-Nancy, France; 19 Department of Medical Oncology, Institut de Cancérologie Jean-Godinot, 1 Rue du Général Koenig, 51100 Reims, France; 20 Department of Medical Oncology, Centre Henri Becquerel, Rue d’Amiens, 76000 Rouen, France; 21 Department of Research and Development, Unicancer, 101 Rue de Tolbiac, 75654 Paris, France; 22 Academic Department of Radiation Oncology, Centre Oscar Lambret, 3 Rue Frédéric Combemale, 59000 Lille, France; 23 Department of Medical Oncology, Centre Léon Bérard, 28 Promenade Léa et Napoléon Bullukian, 69008 Lyon, France

**Keywords:** Metastasis, Outcomes research, Neurological manifestations, Breast cancer

## Abstract

**Background:**

Metastatic breast cancer (MBC) behaviour differs depending on hormone receptors (HR) and human epidermal growth factor receptor (HER2) statuses.

**Methods:**

The kinetics of central nervous system (CNS) metastases (CNS metastasis-free survival, CNSM-FS) and subsequent patient’s prognosis (overall survival, OS) according to the molecular subtype were retrospectively assessed in 16703 MBC patients of the ESME nationwide multicentre MBC database (Kaplan–Meier method).

**Results:**

CNS metastases occurred in 4118 patients (24.6%) (7.2% at MBC diagnosis and 17.5% later during follow-up). Tumours were HER2−/HR+ (45.3%), HER2+/HR+ (14.5%), HER2+/HR− (14.9%) and triple negative (25.4%). Median age at CNS metastasis diagnosis was 58.1 years (range: 22.8–92.0). The median CNSM-FS was 10.8 months (95% CI: 16.5–17.9) among patients who developed CNS metastases. Molecular subtype was independently associated with CNSM-FS (HR = 3.45, 95% CI: 3.18–3.75, triple-negative and HER2−/HR+ tumours). After a 30-month follow-up, median OS after CNS metastasis diagnosis was 7.9 months (95% CI: 7.2–8.4). OS was independently associated with subtypes: median OS was 18.9 months (HR = 0.57, 95% CI: 0.50–0.64) for HER2+/HR+ , 13.1 months (HR = 0.72, 95% CI: 0.65–0.81) for HER2+/HR−, 4.4 months (HR = 1.55, 95% CI: 1.42–1.69) for triple-negative and 7.1 months for HER2−/HR+ patients (*p* <0.0001).

**Conclusions:**

Tumour molecular subtypes strongly impact incidence, kinetics and prognosis of CNS metastases in MBC patients.

**Clinical trial registration:**

NCT03275311.

## Background

Breast cancer is the second most frequent cancer affecting women, and the second most common cause of central nervous system (CNS) metastases, i.e., brain metastases (BM) or leptomeningeal metastases (LM). In total, 30–50% of patients with metastatic breast cancer (MBC) will develop BM in the course of their disease.^1^ The proportion of patients with BM among MBC patients is even higher when considering asymptomatic BM, as suggested in studies evaluating screening strategies^2,3^ or autopsy studies.^4^ The incidence of both BM^5,6^ and LM^7^ has increased. Reasons are longer survival of MBC patients,^8^ the inability of some drugs to cross the blood–brain barrier, an increased control of the systemic disease by human epidermal growth factor receptor 2 (HER2) targeted therapies^5^ or use of more sensitive diagnosis techniques for CNS metastases.

Identifying patients at risk of developing CNS metastases seems crucial. As asymptomatic CNS metastases are frequent, patients at high risk could benefit from screening strategies in order to improve outcomes by allowing, for example, localised BM treatments in a higher proportion of patients.^2^ Indeed, surgery and radiosurgery are yet the only locoregional treatments with a significant positive impact on survival in patients with BM.^9,10^ Risk factors for BM occurrence include younger age at the time of the breast cancer diagnosis,^11^ shorter disease-free survival^12^ and the presence of lung metastases.^11–13^ The biological characteristics of the initial tumour also have an effect on CNS metastasis occurrence.^6,11,14–16^ Hormone receptor (HR) negativity^12,13,17–19^ and HER2 positivity^11,20^ are indeed important risk factors for BM occurrence. Interestingly, differences regarding the time interval to CNS metastasis occurrence according to the breast cancer subtype have been described, with a shorter time interval from BC diagnosis^19,21^ or MBC^12^ to CNS metastasis occurrence for patients with HR-negative^22^ or triple-negative tumours.^12,19,21^ However, these studies were either mono- or bicentric and included a limited number of patients.

Prognosis of patients with CNS metastases is poor, with a median overall survival (OS) ranging from 4 to 25 months for BM^23,24^ and of <6 months in most published series for LM.^25,26^ It depends on various factors, including the patient’s age^19,27^ and performance status, the time interval between the diagnosis of cancer and that of CNS metastases,^27^ the number of BM,^19^ the treatment^19^ (localised treatment vs. no localised treatment) and the control of the extracranial disease.^28^ The tumour biology status also seems to impact outcome, with prolonged survival after BM diagnosis in HER2-positive tumours^19,22^ or poorer survival after LM diagnosis in patients with HR- or triple-negative tumours.^25,27^

The objectives of this study were to provide new data regarding the risk and kinetics of CNS metastasis occurrence during the course of MBC, to identify clinical and biological features associated with a high risk of developing CNS metastases or a shorter time to CNS metastases and to evaluate the patients’ prognosis.

## Patients and methods

### Study design

We performed a retrospective analysis of the 16,703 MBC patients included in the French *Epidemiological Strategy and Medical Economics* (ESME) research programme, to which 18 French specialised cancer centres are participating. The ESME MBC database (NCT03275311) was established, and is managed by R&D UNICANCER.^29,30^ The database included all adult patients treated in first line for an MBC between January 2008 and December 2014, in one of the participating centres.

### Objectives of the study

The primary objective of this study was to evaluate the time interval between MBC diagnosis (stage IV disease) and the occurrence of CNS metastases (CNS metastasis-free survival, CNSM-FS), according to the breast cancer immunohistochemical subtype. The secondary objectives were to evaluate the time interval between the first breast cancer diagnosis and CNS metastases and OS, and to describe progression-free survival (PFS) and CNS–PFS after CNS metastases.

### Selection criteria

For this study, two populations were identified. The overall population included all ESME MBC database patients. Two patients were excluded from analysis due to inconsistencies within the data (population 1). CNSM-FS was evaluated in this population. To evaluate the prognostics of patients after the occurrence of CNS metastases, population 2 included patients from the ESME MBC database diagnosed with CNS metastases at MBC diagnosis or later during the course of MBC. Of note, as the ESME MBC database did not differentiate intraparenchymal BM from LM, both metastatic sites were merged for the analyses.

### Data collected

For all patients, data were extracted from the ESME MBC database. Oestrogen receptors (ER), progesterone receptors (PR) and HER2 statuses were described both at the time of the primary tumour diagnosis and at the time of MBC diagnosis, as some phenotypic changes can be observed between the primary tumour and metastatic recurrence. The HER2 and HR statuses used for statistical analyses were derived from existing results about metastatic tissue sampling, when available, or, if not available, from the last sampling of early breast cancer.

### Statistical considerations

Categorical variables were reported: the number of unavailable data, number and percentage for each variable modality. For continuous variables, the number of missing data, mean, standard deviation, median and range values were computed. All variables were compared by using the Pearson’s χ^2^ test or Student's *t* test, when appropriate. The incidence and prevalence of CNS metastases were calculated in the whole population and in subgroups according to the HR and HER2 statuses. The incidence rate of CNS metastases was defined as the number of patients diagnosed with CNS metastases per 100 person-years, i.e., among 100 patients from population 1 followed for 1 year.

The CNSM-FS was defined as the time interval between the date of MBC diagnosis and the date of CNS metastasis diagnosis. Patients with CNS metastases occurring after the closing date of study analysis (January 15th, 2016), lost to follow-up or dead without CNS metastases were censored at the closing date of analysis. The CNSM-FS was estimated by using the Kaplan–Meier method, presented as median with its 95% confidence interval (95% CI), and survival rates in percentages, with 95% CIs. Survival estimations were compared with the log-rank test. To evaluate the prognostics of patients with CNS metastases, the time between CNS metastasis occurrence and death from any cause (OS), systemic or CNS progression (PFS) and CNS progression (CNS–PFS) were estimated by using the same methods, with adjustment on the major prognostic factors. Patients alive without events were censored at the closing date of study analysis. To investigate prognostic factors, a multivariate analysis was performed by using the Cox’s proportional hazards regression model with a backward procedure. Hazard ratios with their 95% CIs were calculated to display risk changes. All *p*-values reported were two-sided, and the significance level was set at 5% (*p* < 0.05). Statistical analysis was performed by using the SAS^®^ software (version 9.4).

## Results

### Population analysed

**Fig. 1:**
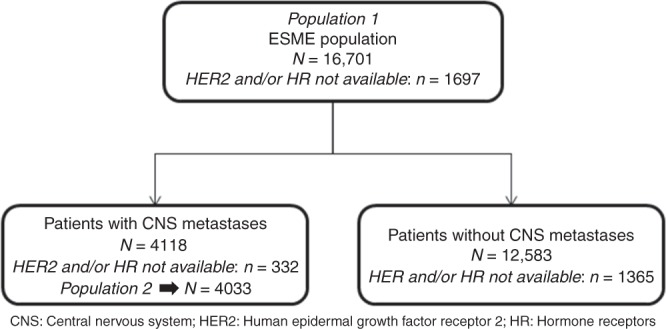
Study flowchart: patients included in the study

A total of 16,703 MBC patients were included in the ESME MBC database (Fig. 1). After exclusion of two patients due to inconsistent data, 16,701 patients were included in the present analysis (population 1). Among them, 4800 had de novo metastatic breast cancer and 11,901 relapsed MBC. After a median follow-up of 42.8 months (95% CI: 42.0–43.7), 4118 patients of population 1 (24.6%) developed CNS metastases. In total, 4033 of them were included in population 2 (85 excluded since diagnosis after 15/01/2016). The characteristics of the two populations of the study are described in Table 1.

**Table 1:** Characteristics of patients selected for the study

	Population 1^a^	Population 2^b^
	*n* = 16,701	*n* = 4033
*Characteristics at breast cancer diagnosis*		
Age at breast cancer diagnosis, median (range), years	54.7 (19.2–98.5)	51.6, 20.3–90.5
Gender		
Female	16 552 (99.1%)	4 007 (99.4%)
Male	149 (0.9%)	26 (0.6%)
Tumour size	*n* = 7646	*n* = 1969
Tx/T0/Tis	363 (4.7%)	100 (5.1%)
T1/T2	4 229 (55.3%)	1 044 (53.0%)
T3/T4	3 054 (39.9%)	825 (41.9%)
Node status	*n* = 7324	*n* = 1901
Nx	318 (4.3%)	77 (4.1%)
N0	3169 (43.3%)	744 (39.1%)
N1/N2/N3	3837 (52.4%)	1080 (56.8%)
Metastatic status	*n* = 16,701	*n* = 16,701
M0	11,901 (71.3%)	2974 (73.7%)
M1	4800 (28.7%)	1059 (26.3%)
Histology	*n* = 15,584	*n* = 3790
Ductal carcinoma	12 537 (80.4%)	3 222 (85.0%)
Lobular carcinoma	2 187 (14.0%)	395 (10.4%)
Other	860 (5.5%)	173 (4.6%)
SBR grade	*n* = 14,779	*n* = 3652
I/II	8308 (56.2%)	1641 (44.9%)
III	6471 (43.8%)	2011 (55.1%)
ER status	*n* = 15,494	*n* = 3800
Negative	3834 (24.7%)	1506 (39.6%)
Positive	11,660 (75.3%)	2294 (60.4%)
PR status	*n* *=* 14,990	*n* *=* 3682
Negative	6375 (42.5%)	2075 (56.4%)
Positive	8615 (57.5%)	1607 (43.6%)
HER2 status	*n* *=* 13,621	*n* *=* 3488
Negative	11,090 (81.4%)	2481 (71.1%)
Positive	2531 (18.6%)	1007 (28.9%)
Tumour biology	*n* *=* 13,498	*n* *=* 3453
HER2−/HR+	8654 (64.1%)	1572 (45.5%)
HER2+/HR+	1480 (11.0%)	504 (14.6%)
HER2−/HR+	1010 (7.5%)	490 (14.2%)
Triple negative	2354 (17.4%)	887 (25.7%)
Radiotherapy delivered on the breast	*n* = 16,654	*n* = 4023
Yes	10,463 (62.8%)	2668 (66.3%)
Systemic treatment	*n* = 16,657	*n* = 4026
Yes	8498 (51.0%)	2463 (61.2%)
Trastuzumab (HER2+ patients)	*n* = 2531	*n* = 1007
Yes	947 (37.4%)	472 (46.9%)
Hormone therapy (HR+ patients)	*n* = 11,947	*n* = 2375
	7354 (61.6%)	1541 (64.9%)
*Characteristics at metastatic disease diagnosis*		
Age at MBC diagnosis, median (range), years	61.2 (19.2–99.4)	56.0 (22.0–91.0)
Time interval from breast cancer diagnosis, median (range), months	35.7 (0.0–657.7)	27.3 (0.0–591.7)
Time interval from breast cancer diagnosis	*n* = 16,656	*n* = 4026
< 6 months	4763 (28.6%)	1053 (26.2%)
6–24 months	2185 (13.1%)	789 (19.6%)
≥ 24 months	9708 (58.3%)	2184 (54.2%)
ER status^c^	*n* = 16,100	*n* = 3906
Positive	11,924 (74.1%)	2292 (58.7%)
PR status^c^	*n* = 15,702	*n* = 3815
Positive	8308 (52.9%)	1515 (39.7%)
HER2 status^c^	*n* = 14,938	*n* = 3707
Positive	2719 (18.2%)	1066 (28.8%)
Tumour biology	*n* = 15,004	*n* = 3708
HER2−/HR+	9533 (63.5%)	1667 (45.0%)
HER2+/HR+	1652 (11.0%)	534 (14.4%)
HER2+/HR−	1168 (7.8%)	557 (15.0%)
Triple negative	2651 (17.7%)	950 (25.6%)
*Number of metastatic sites*		
Median (range)	1 (0–9)	–
CNS metastases	*n* *=* 16,065	–
	1200 (7.5%)	
Bone metastases	*n* *=* 16,067	–
	9512 (59.2%)	
Lung metastases	*n* *=* 16,065	–
	4103 (25.3%)	
Liver metastases	*n* *=* 16,060	–
	4491 (28.0%)	
Lymph node metastases	*n* *=* 16,066	–
	4478 (27.9%)	
Subcutaneous metastases	*n* *=* 16,065	–
	1834 (11.4%)	
Pleural metastases	*n* *=* 16,067	–
	1789 (11.1%)	
Metastases of other site(s)	*n* *=* 16,068	–
	1727 (10.7%)	

*SBR grade* Scarff–Bloom–Richardson grade, *ER* oestrogen receptor, *PR* progesterone receptor, *HER2* human epidermal growth factor receptor 2, *MBC* metastatic breast cancer, *CNS* central nervous system

^a^Population 1 corresponds to the overall ESME population

^b^Population 2 corresponds to patients diagnosed with CNS metastases before the closing date of the study analyses (January 15th, 2016)

^c^ER, PR and HER2 statuses at MBC diagnosis are defined as follows: status at the metastatic disease diagnosis, if available, or status of the primary tumour

### Characteristics of CNS metastases

With a median follow-up of 42.8 months, CNS metastases occurred in 24.6% of all patients from population 1. The incidence rate of CNS metastases (number of patients diagnosed with CNS metastases per 100 person-years, i.e., among 100 patients from population 1 followed for 1 year) was 21.8% in HER2-positive tumours (compared with 11.1% in HER2-negative tumours), 32.5% in HR-negative tumours (compared with 9.2% in HR-positive tumours) and 32.7% in triple-negative tumours, respectively.

The cumulated incidence rate of CNS metastases at 12 months (after MBC diagnosis) was 8.3% (95% CI: 7.8–8.9), 16.8% (95% CI: 15.0–18.8), 32.4% (95% CI: 29.7–35.4) and 29.8% (95% CI: 27.9–31.8) in patients with HER2−/HR+, HER2+/HR+, HER2+/HR− and triple-negative tumours, respectively. At 24 months, it was 14.4% (95% CI: 13.6–15.2), 29.2% (95% CI: 26.8–31.8), 49.0% (95% CI: 45.7–52.5) and 44.8% (95% CI: 42.3–47.3) in patients with HER2−/HR+, HER2+/HR+, HER2+/HR− and triple-negative tumours (Supplementary Table 1). The incidence continued increasing for all tumour subtypes, with no sign of flattening of the incidence over time (cumulated incidence rates up to 36.9%, 53.5%, 72.6% and 71.3% in patients with HER2−/HR+, HER2+:HR+, HER2+/HR− and triple-negative tumours).

The incidence rate of CNS metastases was 13.2% in patients with de novo MBC, and 10.9% in patients with relapse MBC. Among the 4118 patients diagnosed with CNS metastases, 1200 (29.1%) presented with CNS metastases at the time of the initial diagnosis of metastatic disease (isolated CNS metastases in 43.1% of cases). In the overall population, the proportion of patients diagnosed with CNS metastases at metastatic disease diagnosis was 7.2% (4.3%, 9.2%, 17.0% and 13.1% in patients with HER2−/HR+, HER2+/HR+, HER2+/HR− and triple-negative tumours, respectively).

In total, 85 patients from Population 1 were diagnosed with CNS metastases after January 15, 2016 and were excluded from Population 2, which thus included a total of 4033 patients. In this population, the median age at CNS metastases diagnosis was 58.1 years overall; it was 54.1 years for triple-negative patients and 59.9 years for HER2-positive patients (*p* < 0.0001). CNS metastases diagnosis was based on symptoms in 70.7% of patients, and on systematic imaging in 29.3% of patients (asymptomatic patients). The patients’ performance status at the time of CNS metastases diagnosis was available in 1297 patients (32.1%) and distributed as follows: score 0 in 301, score 1 in 615, score 2 in 263, score 3 in 104 and score 4 in 14 patients. With regard to biological subgroups, among patients with CNS metastases 44.9%, 14.4%, 15.0% and 25.6% had an HR+/HER2− HER2+/HR+, HER2+/HR− and triple-negative tumour, respectively.

#### Risk factors for CNSM occurrence and CNSM-FS (population 1, *n* = 16,701)

In population 1 (*n* = 16,701), 4118 patients (24.6%) developed CNS metastases. Among them, 1200 (7.2%) had CNS metastases at the time of MBC diagnosis, while 2918 developed them during the course of MBC, with a median time interval of 17.0 months (95% CI: 16.5–17.9) after MBC diagnosis. The proportion of patients with CNS metastases diagnosed at MBC diagnosis or later during the course of MBC is reported in Supplementary Table 2. Overall, the CNSM-FS (defined as the time interval between the date of MBC diagnosis and the date of CNS metastasis diagnosis) was 10.8 months (95% CI: 10.2–11.5) among patients who developed CNS metastases. The 6-, 12-, 24- and 48-month CNSM-FS rates were 61.5%, 47.2%, 25.3% and 6.4%, respectively, among patients who developed CNS metastases.

**Table 2:** Univariate and multivariate analyses of CNSM-FS

	Univariate analysis			Multivariate analysis *n* = 11,313	
Parameter	Hazard ratio (95% CI)	Median CNSM-FS (months)	*P*-value	Hazard ratio (95% CI)	*P*-value
*SBR grade*			<0.0001		**<0.0001**
I/II	1	NR (90.8–NR)		1	
III	1.96 (1.84–2.10)	52.9 (48.7–57.7)		1.27 (1.18–1.38)	
*Histological subtype*			<0.0001		
Ductal carcinoma	1	77.8 (73.3–90.8)			
Lobular carcinoma	0.67 (0.60–0.74)	NR (78.2–NR)			
Other	0.84 (0.72–0.98)	NR (NR–NR)			
*Age at MBC diagnosis*			<0.0001		**<0.0001**
<50	1	59.6 (55.5-66.7)		1	
50–70	0.76 (0.71–0.82)	76.5 (73.3–85.9)		0.94 (0.87–1.02)	
>70	0.43 (0.39–0.48)	NR (NR–NR)		0.66 (0.59–0.75)	
*MFI (months)*			<0.0001		**<0.0001**
<6	1	83.3 (73.7–NR)		1	
6–24	2.57 (2.35–2.83)	33.5 (29.1–41.1)		1.44 (1.20–1.73)	
≥24	1.00 (0.93–1.08)	91.1 (86.0–NR)		0.96 (0.80–1.15)	
*Number of metastatic sites at MBC diagnosis*			<0.0001		**<0.0001**
<3	1	NE (86.0–NE)		1	
≥3	2.14 (2.00–2.29)	53.8 (47.9–61.1)		2.16 (1.99–2.34)	
*Tumour biology*^a^			<0.0001		**<0.0001**
HER2−/HR+	1	NR (91.1–NR)		1	
HER2+/HR+	2.01 (1.83–2.22)	61.7 (51.7–74.1)		1.33 (0.89–1.99)	
HER2+/HR−	3.69 (3.35–4.06)	24.9 (22.7–28.9)		2.01 (1.29–3.15)	
Triple negative	3.45 (3.18–3.75)	29.9 (27.0–33.1)		1.57 (1.25–1.97)	
*HER2 status*^a^			<0.0001		
Negative	1	NR (85.9–NR)			
Positive	1.93 (1.80–2.07)	40.8 (37.3–47.5)			
*ER status*^a^			<0.0001		
Positive	1	NR (90.8–NR)			
Negative	3.07 (2.88–3.28)	29.0 (26.3–32.5)			
*PR status*^a^			<0.0001		
Positive	1	NR (91.1–NR)			
Negative	2.20 (2.06–2.34)	53.8 (47.9–61.1)			
*HR status*^a^			<0.0001		
Positive	1	NE (90.8–NE)			
Negative	3.19 (2.98–3.41)	27.0 (23.6–29.5)			
*Previous systemic treatment (per os)*			<0.0001		
No	1	86.0 (80.7–NR)			
Yes	1.63(1.35–1.97)	71.4 (67.5–78.6)			
*Previous systemic treatment (IV)*			<0.0001		**0.0020**
No	1	NR (86.0–NR)		1	
Yes	1.65 (1.54–1.75)	71.4 (67.5–78.6)		1.22 (1.08–1.39)	
*Previous radiation therapy*			<0.0001		**0.0119**
No	1	85.7 (75.6–NR)		1	
Yes	1.20 (1.13–1.29)	85.9 (77.8–NR)		1.22 (1.04–1.42)	

*CNSM-FS* central nervous system metastases-free survival, *ER* oestrogen receptor, *HER2* human epidermal growth factor receptor 2, *IV* intravenous, *MFI* metastases-free interval, *NR* not reached, *PR* progesterone receptor, *SBR grade* Scarff–Bloom–Richardson grade

^a^At MBC diagnosis, statuses defined as follows: status at the metastatic disease diagnosis, if available, or status of the primary tumour,

Bold values indicate statistical significance p < 0.05

**Fig. 2:**
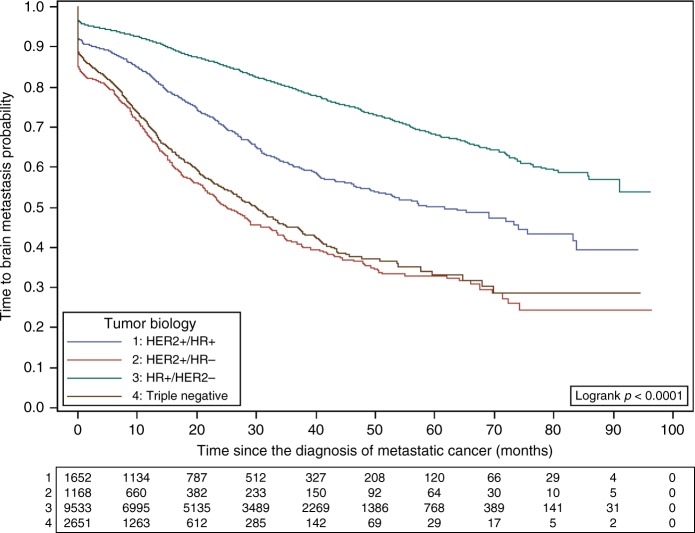
CNSM-FS according to the tumour biology

The results of the univariate CNSM-FS analysis are provided in Table 2. Among patients who developed CNS metastases, the molecular subtype (at MBC diagnosis) was significantly associated with CNSM-FS: HER2−/HR+ 15.1 (95% CI: 14.3–16.1), HER2+/HR+ 12.8 (95% CI: 11.5–14.4), HER2+/HR− 8.5 (95% CI: 6.8–9.6) and triple-negative 5.8 months (95% CI: 4.8–6.7) (*p* < 0.001) (Fig. 2). HER2 positivity and HR, ER and PR negativity were also associated with a shorter CNSM-FS when considered independently (Table 2 and Supplementary Fig. 1).

In multivariate analysis, molecular subtype was independently associated with CNSM-FS: hazard ratios were 2.01 (95% CI: 1.29–3.15, *p* = 0.0021) and 1.57 (95% CI: 1.25–1.97, *p* < 0.0001) for HER2+/HR− and triple-negative tumours, respectively (compared with HER2−/HR+ tumours). A higher histoprognostic grade, longer metastases-free interval ([6–24 [vs. <6 months), younger age at MBC diagnosis ( <50 vs. >70), higher number of metastatic sites at MBC diagnosis ( ≥3 vs. <3) and having received previous intravenous systemic therapy or radiation therapy were also associated with a shorter CNSM-FS (Table 2).

#### Prognostic of patients with CNS metastases (population 2, *n* = 4033)

Following CNS metastases diagnosis, 2.3% of patients underwent a neurosurgical resection of the lesions, 10.5% received a stereotactic radiation therapy, 45.2% whole-brain radiation therapy, 59.3% a systemic treatment and 16.2% best supportive care only.

#### Overall survival

With a 30-month median follow-up (95% CI: 28.0–32.0) after CNS metastases diagnosis (population 2), 2983 patients (74%) had died. Median OS after CNS metastases diagnosis was 7.9 months (95% CI: 7.2–8.4). The 6-, 12-, 24- and 48-month survival rates were 56.3%, 37.7%, 22.1% and 8.0%, respectively.

Median OS was 7.1 months (95% CI: 6.3–7.9) for HER2−/HR+, 18.9 months (95% CI: 15.0–23.0) for HER2+/HR+, 13.1 months (95% CI: 11.7–15.2) for HER2+/HR− and 4.4 months (95% CI: 4–4.8) for triple-negative tumours (*p* < 0.0001).

**Fig. 3:**
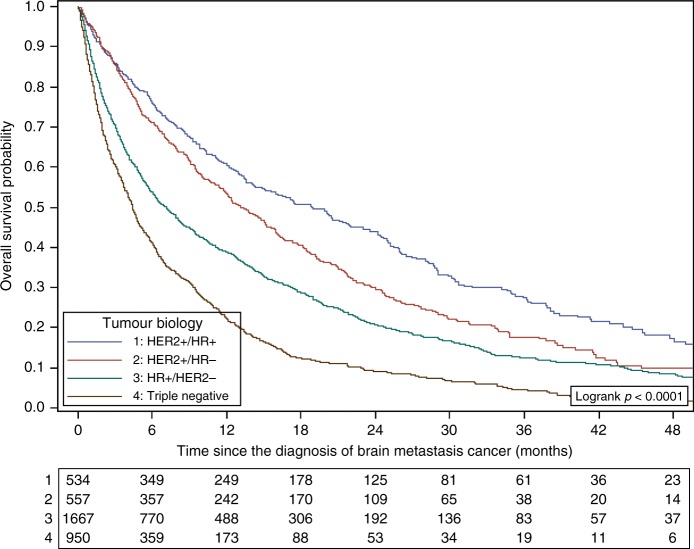
OS after CNS metastases diagnosis according to the tumour biology

The results of the univariate analysis of OS are provided in Table 3, Fig. 3 and Supplementary Fig. 2. In multivariable analysis, the molecular subtype was independently associated with OS: compared with HER2−/HR+ tumours, hazard ratios were 0.90 (95% CI: 0.80–1.02, *p* < 0.0001), 0.63 (95% CI: 0.55–0.71, *p* < 0.0001) and 1.87 (95% CI: 1.70–2.06, *p* < 0.0001) for HER2+/HR−, HER2+/RH+ and triple-negative tumours, respectively. An older age, symptomatic CNS metastases, a longer time interval between breast cancer and CNS metastases diagnosis, a higher number of metastatic sites and a higher number of previous chemotherapy lines were also associated with shorter OS (Table 3). Of note, performance status, a known prognostic factor in patients with CNS metastases, was not included in the multivariate model due to a high number of missing data (67.8%). Amongst patients with CNS metastases at initial diagnosis of metastatic disease, patients with isolated CNS metastases showed a longer OS compared with patients diagnosed concomitantly with other metastatic site(s) (hazard ratio 0.86, 95% CI: 0.76–0.99, *p* = 0.035).

**Table 3:** Prognostic factors of OS in patients diagnosed with CNS metastases

	Univariate analysis			Multivariate analysis *n* = 3496	
Parameter	Hazard ratio (95% CI)	Median OS (months)	*P*-value	Hazard ratio (95% CI)	*P*-value
*Age at CNSM diagnosis*			<0.0001		**<0.0001**
<50	1	10.5 (9.4–12.0)		1	
50–70	1.18 (1.09–1.28)	7.5 (6.9–8.5)		1.20 (1.09–1.31)	
>70	1.60 (1.43–1.79)	4.5 (4.0–5.3)		1.72 (1.52–1.94)	
*Performance status at CNSM diagnosis*					
0	1	18.5 (14.7–22.1)			
1	1.48 (1.23–1.79)	9.5 (7.9–11.6)			
2	1.80 (1.44–2.24)	5.4 (4.0–8.8)			
3	3.40 (2.57–4.51)	2.7 (2.0–3.7)			
4	2.65 (1.35–5.21)	5.1 (2.2–14.2)			
*Symptoms at CNSM diagnosis*			<0.0001		**0.0001**
Present	1	7.4 (6.8–8.0)		1	
Absent	0.81 (0.75–0.88)	10.1 (9.0–11.7)		0.84 (0.77–0.92)	
*Time interval between breast cancer and CNSM diagnosis (months)*			<0.0001		**0.0002**
<9	1	9.4 (8.3–10.1)		1	-
9–18	1.29 (1.18–1.42)	6.7 (6.0–8.3)		1.08 (0.97–1.21)	
≥18	1.31 (1.21–1.43)	6.3 (5.6–7.2)		0.84 (0.75–0.95)	
*Tumour biology*^a^			<0.0001		**<0.0001**
HER2−/HR+	1	7.1 (6.3–7.9)		1	–
HER2+/HR+	0.57 (0.50–0.64)	18.9 (15.0–23.0)		0.63 (0.55–0.72)	<0.0001
HER2+/HR−	0.72 (0.65–0.81)	13.1 (11.7–15.2)		0.86 (0.77–0.97)	<0.0001
Triple negative	1.55 (1.42–1.69)	4.4 (4.0–4.8)		1.88 (1.71–2.07)	<0.0001
*HER2 status*^a^			<0.0001		
Positive	1	15.1 (13.5–17.3)			
Negative	1.81 (1.66–1.97)	5.7 (5.3–6.2)			
*ER status*^a^			<0.0001		
Positive	1	9.1 (8.1–9.8)			
Negative	1.28 (1.19–1.38)	6.4 (5.9–7.1)			
*PR status*^a^			<0.0001		
Positive	1	8.5 (7.7–9.5)			
Negative	1.17 (1.08–1.26)	7.1 (6.5–7.9)			
*HR status*^a^			<0.0001		
Positive	1	9.0 (8.1–9.8)			
Negative	1.30 (1.20–1.40)	6.4 (5.8–7.0)			
*Number of metastatic sites*			<0.0001		**<0.0001**
<3	1	12.1 (10.8-13.1)		1	
≥3	1.52 (1.41–1.65)	6.0 (5.6–6.5)		1.46 (1.34–1.60)	
*Number of previous chemotherapy lines*			<0.0001		**<0.0001**
<3	1	10.0 (9.4–10.7)		1	–
≥3	1.91 (1.76–2.08)	4.3 (3.9–4.8)		2.01 (1.79–2.27)	<0.0001

*OS* overall survival, *CNS* central nervous system, *CNSM* CNS metastases, *ER* oestrogen receptor, *PR* progesterone receptor, *HER2* human epidermal growth factor receptor 2

^a^At MBC diagnosis, statuses defined as follows: status at the metastatic disease diagnosis, if available, or status of the primary tumour

Bold values indicate statistical significance p < 0.05

#### Progression-free survival

The median PFS after diagnosis of CNS metastases was 3.3 months (95% CI: 3.1–3.4). The PFS rates at 6, 12, 24 and 48 months were 28.8%, 11.5%, 3.5% and 0.9%, respectively. In multivariable analysis, an older age at CNS metastases diagnosis, a longer time interval between breast cancer and CNS metastases diagnosis, a higher number of metastatic sites and a higher number of previous chemotherapy lines were independently associated with a shorter PFS. The tumour biology also had a significant effect on PFS, triple-negative tumours being associated with a shorter PFS and HER2-positive tumours with a longer PFS (Supplementary Table 3). Among patients with CNS metastases at initial diagnosis of metastatic disease, a longer PFS was reported for patients with isolated CNS metastases compared with those with other metastatic site(s) (hazard ratio 0.88, 95% CI: 0.78–0.99, *p* = 0.034).

#### CNS–PFS

The median CNS–PFS was 5.5 months (95% CI: 5.2–5.8). The CNS–PFS rates at 6, 12, 24 and 48 months were 47.1%, 26.2%, 11.5% and 3.6%, respectively. In multivariable analysis, factors independently associated with a shorter CNS–PFS were the same as for PFS. As for PFS, triple-negative tumours were associated with a shorter CNS–PFS (Supplementary Table 4). The CNS–PFS was not significantly associated with de novo diagnosis compared with relapsed MBC (median PFS: 6.1 months and 5.3 months, respectively, *p* = 0.052).

### Subgroup of patients with HER2-positive tumours

Among the 2 531 patients (from population 1) with a HER2-positive tumour, 1027 developed CNS metastases. The incidence rate of CNS metastases was 16.6% in patients with de novo MBC (*n* = 1044, 41.2%) and 27.2% in patients with relapse MBC (*n* = 1487, 58.7%). Amongst the 1027 patients with CNS metastases, the median CNSM-FS of 38.3 months (95% CI: 34.0–43.2) (median time interval among the 1027 patients with CNS metastases: 10.2 months, 95% CI: 9.0–11.4). In multivariable analysis, an older age at CNS metastases diagnosis, a longer time interval between breast cancer diagnosis and MBC, a higher number of metastatic sites, HR negativity and the administration of a previous HER2-targeted therapy were independently associated with a shorter CNSM-FS (Supplementary Table 5).

After a median follow-up of 29.9 months (95% CI: 27.6–33.0) after CNS metastasis diagnosis, 649 patients (64.5%) had died. The median OS was 15.2 months (95% CI: 13.5–17.4), and survival rates at 6, 12, 24 and 48 months were 73.9%, 57.1%, 36.4% and 13.6%, respectively.

In multivariable analysis, we found that an older age, HR negativity, a higher number of metastatic sites, a higher number of previous chemotherapy lines and no administration of a previous HER2-targeted therapy were prognostic factors associated with a shorter OS (Supplementary Table 6).

## Discussion

We show, in a large multicentre real-world database of MBC patients, that breast cancer molecular subtype strongly impacts the occurrence, kinetics and prognosis of CNS metastases.

Only few data are available regarding prevalence and incidence rates of CNS metastases in breast cancer patients, as, to our knowledge, no nationwide reporting system for breast cancer patients diagnosed with CNS metastases exists. The data come from autopsy studies and population-based studies with various methodologies. Our analysis of the EMSE MBC database provides an estimate of the cumulated incidence rate of CNS metastases among MBC patients of 24.6%. At the time of metastatic disease diagnosis, 7.2% of patients were diagnosed with CNS metastases (12.3% in patients with HER2+ tumours). In a population-based study from the “Surveillance, Epidemiology and End Results” (SEER) database, 614/7575 patients (8.1%) with de novo MBC were diagnosed with BM at MBC diagnosis (105 with isolated BM and 509 with other metastatic sites).^31^ In a prospective cohort study from the “National Comprehensive Cancer Network” (NCCN) including 3394 HER2+ patients, BM was present at the first recurrence in 20 and 13% of patients with a HER2+/HR− and a HER2+/RH+ tumours, respectively.^32^

We found an overrepresentation of HER2-positive, HR-negative and triple-negative tumours among MBC patients diagnosed with CNS metastases, suggesting that tumour biology impacts the risk of CNS involvement in breast cancer. Indeed, an increased risk of BM was reported in previous studies for HER2-positive^11,20,33,34^ and HR-negative^17,19,21,22,35–37^ patients. In our study, the cumulated incidence rate of CNS metastases was 21.8% in HER2-positive tumours (compared with 11.1% in HER2-negative tumours), 32.5% in HR-negative tumours (compared with 9.2% in HR-positive tumours) and 32.7% in triple-negative tumours, respectively, consistent with previous literature data.^37–39^ HER2-positive, HR-negative and triple-negative tumours were reported respectively in 29.4%, 40.6% and 25.6% of patients with CNS metastases. This compares with 18.8%, 25.5% and 17.7% of patients in the whole ESME database and with 16.6%, 26.4% and 19.5% in a series of 11 011 unselected stage I–III breast cancer patients.^40^ These proportions in patients with CNS metastases are consistent with previous data for HR-negative^19,35,36,41,42^ and triple-negative tumours,^15,21,41,42^ but not for HER2-positive tumours. Indeed, previous reports have described higher proportions (34.8–43.1%) of HER2-positive tumours amongst patients with BM.^21,41,42^ In our study, patients were included more recently, and HER2-targeted therapies might have been used more often in the early and metastatic phase of the disease. This could have led to a selection bias favouring HER2-negative tumours among patients with CNS metastases. However, this hypothesis must be considered with caution, as some studies have reported an increased risk of BM in patients treated with trastuzumab during the adjuvant or metastatic phase.^5,43,44^ If confirmed, this could explain our finding of an increased incidence rate of CNS metastases in patients with relapsed MBC (27.2%) compared with patients with de novo MBC (16.6%). Yet, the impact of previous treatment with trastuzumab on the risk of CNS metastases occurrence is debated, as these results were not reproduced in other studies.^12,34,45^ Of note, the fact that most patients with CNS metastases have a HER2−/HR+ tumour (45.3%) despite an increased risk of CNS metastases in patients with a HER2-positive and/or a HR-negative tumour is linked with the fact that the incidence of HER2−/HR+ tumours is higher.

In our study, the median time interval between MBC diagnosis and the occurrence of CNS metastases was 10.8 months amongst patients who developed CNS metastases, compared with 10–19 months in previous studies.^12,21,39^ Because patients with CNS metastases occurring after the closing date of study analysis, lost to follow-up or dead without CNS metastases were censored at the closing date of analysis, this time interval is quite prolonged as compared with reported survivals of MBC patients (37.2 months in the ESME MBC database^30^), suggesting an increased risk of CNS metastases in long MBC survivors (consistently with clinical observation in daily practice). We confirmed a significant impact of tumour biology, HER2 positivity and HR negativity associated with a shorter CNSM-FS.^12,21,34^ These patients at high risk of CNS metastases could potentially benefit from screening strategies, as CNS metastases can be asymptomatic, in order to improve the patients’ outcomes by allowing localised BM treatments in a higher proportion of patients.^2^ Indeed, surgery and radiosurgery are yet the only locoregional treatments with a significant positive impact on survival.^9,10^ Prospective studies in this selected population of patients should evaluate the prognostic impact of such strategy. In patients with a HER2-positive tumour, our results show a negative impact of HR negativity on CNSM-FS and on survival following CNS metastases. This result confirms previous data showing a higher risk of CNS metastases^34^ and a shorter time interval to CNS involvement^12^ in patients with a HR−/HER2+ tumour compared with HR+/HER2+ patients. Moreover, we found that patients previously treated with a HER2-targeted therapy had a shorter CNSM-FS compared with those who did not receive such treatment. Previous studies have reported an increased risk of BM in patients treated with trastuzumab during the adjuvant or metastatic phase^5,43,45^ This could be due to an increase control of the systemic disease by trastuzumab, resulting in an improved “systemic” survival while CNS involvement is not prevented because of the low penetration of trastuzumab in the brain. Further studies are warranted to better clarify these hypotheses.^12,34,46^ Moreover, it must be noted that once the BM has occurred, a number of studies have demonstrated a clinical efficacy of systemic treatment including HER2-targeted therapies.^47,48^

The prognosis of patients with CNS metastases is poor. In our study, the median OS after the CNS metastasis diagnosis was 7.9 months, lower than that reported in previous breast cancer-related BM series (median OS around 14 months^19,24^), possibly linked to differences in the inclusion criteria (in the Sperduto’s study, only patients referred for radiation therapy were included, whereas this database includes all patients with BM, whatever the treatment modalities). Another important difference is the fact that we included patients with both BM and LM. Indeed, while 4–16-month survivals have been reported in patients with BM,^24^ survival is <5 months in patients with LM.^7,22,46^ Our results confirmed that survival differs significantly according to the tumour biology, HR-negative, HER2-negative or triple-negative tumours being classically associated with poorer survival.^19,21,39,41,46,49,50^ These biological features have been included in the modified-Breast Graded Prognostic Assessment (GPA) score that aims at predicting survival of MBC patients with BM.^51^ In patients with a HER2-positive tumour, our study shows that OS after CNS metastasis diagnosis is negatively impacted by HR negativity. This is consistent with the results of the study based on the SEER data published by Kim et al.: the median OS was 10 months in patients with a HER2+/HR− tumour compared with 23 months in those with a HER2+/HR+ tumour.^49^

We report that a low proportion of patients treated with surgery (2.3%) or stereotactic radiation therapy (10.5%) following CNS metastases should be investigated further in another study.

Our study has some limitations. First, because the database was not specifically designed to study CNS metastases, it was not possible to distinguish intraparenchymal BM from LM. Second, because of unavailable data, some parameters could not be included in the multiparameter analysis of OS despite their demonstrated prognostic value. Indeed, for example, the number of BM was not described in the ESME MBC database, while it was shown to be prognostic in these patients. Also, it must be acknowledged that the proportion of patients diagnosed with CNS metastases depends on the duration of the patients’ follow-up. This could cause a bias for patients who are still alive at the time of the study analysis (representing 45.1% of patients), as it is possible that these patients have or will be diagnosed with CNS metastases after this date. Finally, the results of our survival analyses must be considered with caution, in particular regarding the prognostic value of parameters describing the initial diagnosis, as our population is only composed of metastatic patients.

In conclusion, in this large multicentre real-life study including more than 16,000 MBC patients and over 4000 patients with CNS metastases, we found that the breast cancer molecular subtype strongly impacts the occurrence and kinetics of CNS metastases and the patients’ prognosis. HR-negative, HER2-positive and triple-negative tumours are overrepresented in patients developing CNS metastases, supporting a higher risk of CNS metastases in these biological subtypes. Thus, patients with HR-negative and/or HER2-positive tumours could represent a population of choice for clinical trials evaluating treatment strategies for CNS metastases, as well as screening or preventive approaches.

## Supplementary information

Supplementary figures and tables

## Data Availability

The analysis data set will be made available upon request.
