# Supplementary material for: Impact of breast cancer molecular subtypes on the incidence, kinetics and prognosis of central nervous system metastases in a large multicentre real-life cohort
Source: Br J Cancer. 2019 Nov 13;121(12):991–1000. doi: 10.1038/s41416-019-0619-y (PMC6964671; doi:10.1038/s41416-019-0619-y)
Supplement: Supplementary file 1 — Supplementary figures and tables [file 41416_2019_619_MOESM1_ESM.docx]

**Supplementary Figure 1.** CNSM-FS according to the (A) HER2 status, (B) HR status, (C) ER status or (D) PR status

**
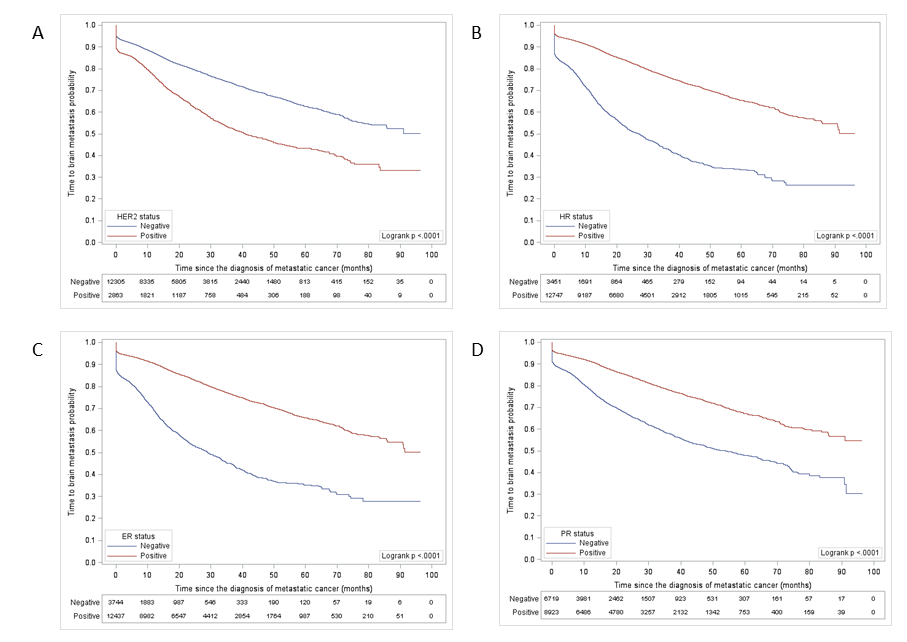
**

**Supplementary Figure 2.** OS after CNS metastases diagnosis according to the (A) HER2 status, (B) HR status, (C) ER status or (D) PR status

**
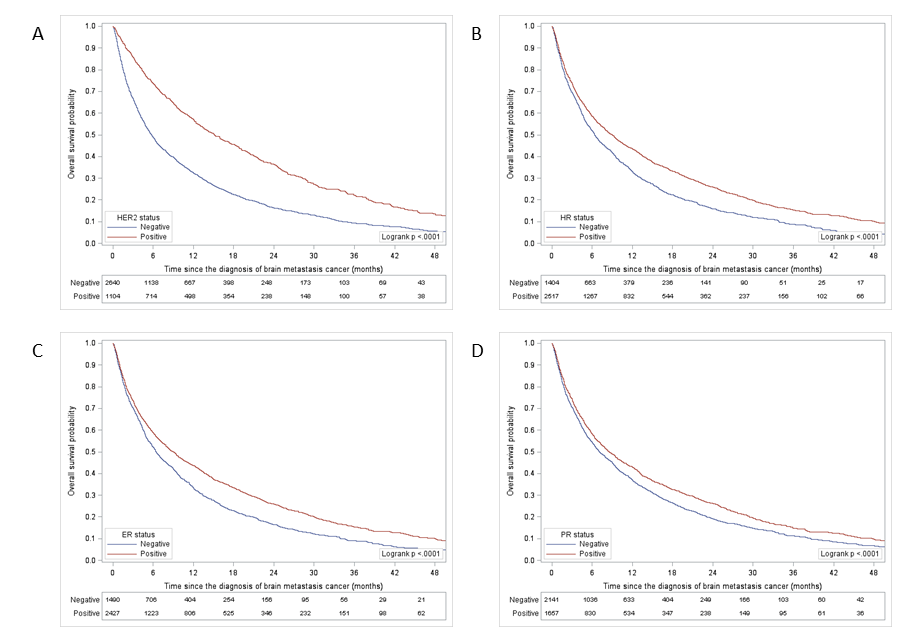
**

**Supplementary Table 1.** Cumulated incidence rate (%) of CNS metastases (with 95% CIs) according to the tumor immunohistochemical subtype.

| **Duration** | **HER2-/HR+** | **HER2+/HR+** | **HER2+/HR-** | **Triple-negative** |
| --- | --- | --- | --- | --- |
| 1 year | 8.3 (7.8-8.9) | 16.8 (15.0-18.8) | 32.4 (29.7-35.4) | 29.8 (27.9-31.8) |
| 2 years | 14.4 (13.6-15.2) | 29.2 (26.8-31.8) | 49.0 (45.7-52.5) | 44.8 (42.3-47.3) |
| 3 years | 20.2 (19.2-21.3) | 39.5 (36.6-42.5) | 58.6 (55.0-62.3) | 54.8 (51.7-58.0) |
| 4 years | 26.1 (24.9-27.5) | 45.4 (42.1-48.8) | 64.0 (60.1-67.9) | 62.8 (58.9-66.6) |
| 5 years | 31.8 (30.2-33.5) | 49.8 (46.1-53.7) | 67.1 (62.9-71.2) | 66.9 (62.0-71.8) |
| 6 years | 36.9 (34.7-39.1) | 53.5 (49.0-58.1) | 72.6 (67.4-77.7) | 71.3 (64.9-77.5) |

**Supplementary Table 2.** Timing of CNS metastases occurrence in the course of MBC.

| **CNS metastases:** | **HER2-/HR+** | **HER2+/HR+** | **HER2+/HR-** | **Triple-negative** | **Total** |
| --- | --- | --- | --- | --- | --- |
| Total number | 1714 | 548 | 564 | 960 | 3786^a^ |
| At MBC diagnosis | 406 (23.7%) | 152 (27.7) | 198 (35.1) | 348 (36.3) | 1104 (29.2%) |
| Later during MBC | 1308 (76.3%) | 396 (72.3) | 366 (64.9) | 612 (63.7) | 2682 (70.8) |

^a^ analyses were not performed on the 4118 patients from *Population 1* as information on HER2 and/or HR was not available for 332 of them.

**Supplementary Table 3.** Prognostic factors of PFS in patients diagnosed with CNS metastases.

|  | **Univariate analysis** | | | **Multivariate analysis** | |
| --- | --- | --- | --- | --- | --- |
| **Parameter** | **Hazard-ratio (95% CI)** | **Median PFS (months)** | ***P*-value** | **Hazard-ratio**  **(95% CI)** | ***P*-value** |
| **Age at CNSM diagnosis** |  |  | 0.0015 |  | **0.0003** |
| <50 | 1 | 3.6 (3.4-4.0) |  | 1 |  |
| [50-70[ | 1.06 (0.99-1.15) | 3.2 (3.0-3.4) |  | 1.06 (0.98-1.14) |  |
| >70 | 1.20 (1.09-1.33) | 2.8 (2.5-3.1) |  | 1.25 (1.12-1.40) |  |
| **Performance status at CNSM diagnosis** |  |  | <0.0001 |  |  |
| 0 | 1 | 4.7 (3.8-5.6) |  |  |  |
| 1 | 1.23 (1.05-1.42) | 3.2 (2.9-3.5) |  |  |  |
| 2 | 1.39 (1.16-1.67) | 2.6 (2.2-3.1) |  |  |  |
| 3 | 2.13 (1.65-2.74) | 2.0 (1.6-2.5) |  |  |  |
| 4 | 2.06 (1.18-3.61) | 2.7 (1.7-5.1) |  |  |  |
| **Symptoms at CNSM diagnosis** |  |  | 0.0003 |  | **0.0003** |
| Present | 1 | 3.1 (3.0-3.3) |  | 1 |  |
| Absent | 0.87 (0.81-0.94) | 3.7 (3.4-4.1) |  | 0.87 (0.80-0.94) |  |
| **Time interval between breast cancer and CNSM diagnosis (months)** |  |  | <0.0001 |  | **<0.0001** |
| < 9 | 1 | 3.6 (3.4-4.0) |  | 1 |  |
| [9-18[ | 1.33 (1.22-1.45) | 2.9 (2.7-3.1) |  | 1.15 (1.04-1.27) |  |
| ≥ 18 | 1.32 (1.22-1.42) | 2.9 (2.8-3.2) |  | 0.91 (0.81-1.01) |  |
| **Tumor biology^a^** |  |  | <0.0001 |  | **<0.0001** |
| HER2- / HR+ | 1 | 3.0 (2.9-3.3) |  | 1 |  |
| HER2+ / HR+ | 0.77 (0.69-0.85) | 4.7 (4.2-5.3) |  | 0.83 (0.74-0.92) |  |
| HER2+ / HR- | 0.82 (0.74-0.90) | 4.8 (4.2-5.1) |  | 0.93 (0.84-1.03) |  |
| Triple negative | 1.42 (1.31-1.55) | 2.4 (2.2-2.6) |  | 1.63 (1.49-1.78) |  |
| **HER2 status^a^** |  |  | <0.0001 |  |  |
| Positive | 1 | 4.7 (4.3-5.0) |  |  |  |
| Negative | 1.42 (1.32-1.53) | 2.8 (2.7-2.9) |  |  |  |
| **ER status^a^** |  |  | <0.0001 |  |  |
| Positive | 1 | 3.4 (3.2-3.6) |  |  |  |
| Negative | 1.19 (1.11-1.28) | 3.0 (2.8-3.3) |  |  |  |
| **PR status^a^** |  |  | 0.0003 |  |  |
| Positive | 1 | 3.4 (3.2-3.7) |  |  |  |
| Negative | 1.13 (1.06-1.21) | 3.1 (2.9-3.3) |  |  |  |
| **HR status^a^** |  |  | <0.0001 |  |  |
| Positive | 1 | 3.4 (3.2-3.6) |  |  |  |
| Negative | 1.21 (1.13-1.30) | 3.0 (2.8-3.2) |  |  |  |
| **Number of metastatic sites** |  |  | <0.0001 |  | **<0.0001** |
| < 3 | 1 | 4.6 (4.3-4.9) |  | 1 |  |
| ≥ 3 | 1.51 (1.41-1.62) | 2.8 (2.7-2.9) |  | 1.42 (1.31-1.54) |  |
| **Number of previous chemotherapy lines** |  |  | <0.0001 |  | **<0.0001** |
| < 3 | 1 | 3.7 (3.5-3.9) |  | 1 |  |
| ≥ 3 | 1.72 (1.59-1.85) | 3.2 (3.0-3.3) |  | 1.68 (1.51-1.87) |  |

PFS: Progression-Free Survival; CNS: Central Nervous System; CNSM: CNS Metastases; ER: Estrogen Receptor; PR: Progesterone Receptor; HER2: Human Epidermal growth factor Receptor 2; IV: Intra-Venous ^a^ at MBC diagnosis, statuses defined as follows: status at the metastatic disease diagnosis, if available, or status of the primary tumor

**Supplementary Table 4.** Prognostic factors of CNS-PFS in patients diagnosed with CNS metastases.

|  | **Univariate analysis** | | | **Multivariate analysis** | |
| --- | --- | --- | --- | --- | --- |
| **Parameter** | **Hazard-ratio (95% CI)** | **Median CNS-PFS (months)** | ***P*-value** | **Hazard-ratio**  **(95% CI)** | ***P*-value** |
| **Age at CNSM diagnosis** |  |  | <0.0001 |  | **<0.0001** |
| <50 | 1 | 6.4 (6.0-7.1) |  | 1 |  |
| [50-70[ | 1.08 (1.00-1.17) | 5.4 (5.0-5.8) |  | 1.09 (1.01-1.19) |  |
| >70 | 1.31 (1.18-1.46) | 4.0 (3.5-4.6) |  | 1.44 (1.29-1.62) |  |
| **Performance status at CNSM diagnosis** |  |  | <0.0001 |  |  |
| 0 | 1 | 8.5 (7.4-9.9) |  |  |  |
| 1 | 1.33 (1.13-1.56) | 5.9 (5.1-7.0) |  |  |  |
| 2 | 1.56 (1.28-1.90) | 4.0 (3.3-5.0) |  |  |  |
| 3 | 2.66 (2.04-3.46) | 2.5 (2.0-3.3) |  |  |  |
| 4 | 1.89 (0.97-3.68) | 4.7 (2.2-14.2) |  |  |  |
| **Symptoms at CNSM diagnosis** |  |  | <0.0001 |  | **<0.0001** |
| Present | 1 | 5.3 (5.0-5.7) |  | 1 |  |
| Absent | 0.82 (0.76-0.88) | 6.1 (5.6-6.8) |  | 0.85 (0.78-0.92) |  |
| **Time interval between breast cancer and CNSM diagnosis (months)** |  |  | <0.0001 |  | **0.0003** |
| < 9 | 1 | 6.1 (5.7-6.6) |  | 1 |  |
| [9-18[ | 1.29 (1.18-1.41) | 4.8 (4.3-5.7) |  | 1.11 (1.01-1.24) |  |
| ≥ 18 | 1.25 (1.15-1.35) | 4.8 (4.5-5.2) |  | 0.89 (0.79-0.99) |  |
| **Tumor biology^a^** |  |  | <0.0001 |  | **<0.0001** |
| HER2- / HR+ | 1 | 5.3 (4.9-5.7) |  | 1 |  |
| HER2+ / HR+ | 0.80 (0.71-0.89) | 8.8 (8.0-10.0) |  | 0.86 (0.77-0.96) |  |
| HER2+ / HR- | 0.93 (0.84-1.04) | 6.9 (6.4-7.7) |  | 1.08 (0.97-1.21) |  |
| Triple negative | 1.60 (1.47-1.75) | 3.7 (3.4-4.1) |  | 1.85 (1.6-2.03) |  |
| **HER2 status^a^** |  |  | <0.0001 |  |  |
| Positive | 1 | 7.8 (7.2-8.5) |  |  |  |
| Negative | 1.36 (1.26-1.47) | 4.6 (4.2-4.9) |  |  |  |
| **ER status^a^** |  |  | <0.0001 |  |  |
| Positive | 1 | 6.1 (5.7-6.6) |  |  |  |
| Negative | 1.35 (1.26-1.45) | 4.8 (4.6-5.1) |  |  |  |
| **PR status^a^** |  |  | <0.0001 |  |  |
| Positive | 1 | 6.0 (5.5-6.4) |  |  |  |
| Negative | 1.20 (1.12-1.29) | 5.1 (4.9-5.5) |  |  |  |
| **HR status^a^** |  |  | <0.0001 |  |  |
| Positive | 1 | 6.1 (5.7-6.5) |  |  |  |
| Negative | 1.37 (1.27-1.47) | 4.8 (4.5-5.1) |  |  |  |
| **Number of metastatic sites** |  |  | <0.0001 |  | **<0.0001** |
| < 3 | 1 | 7.4 (6.9-7.9) |  | 1 |  |
| ≥ 3 | 1.41 (1.31-1.51) | 4.7 (4.3-4.9) |  | 1.37 (1.26-1.49) |  |
| **Number of previous chemotherapy lines** |  |  | 0.0012 |  | **<0.0001** |
| < 3 | 1 | 6.3 (6.0-6.7) |  | 1 |  |
| ≥ 3 | 1.62 (1.50-1.76) | 3.8 (3.4-4.1) |  | 1.68 (1.51-1.88) |  |

CNS: Central Nervous System; CNSM: CNS metastases; CNS-PFS: CNS Progression-Free Survival; ER: Estrogen Receptor;
PR: Progesterone Receptor; HER2: Human Epidermal growth factor Receptor 2  ^a^ at MBC diagnosis, statuses defined as follows: status at the metastatic disease diagnosis, if available, or status of the primary tumor.

**Supplementary Table 5.** Univariate and multivariate analyses of CNSM-FS in the subgroup of patients with a HER2-positive tumor.

|  | **Univariate analysis** | | | **Multivariate analysis** | |
| --- | --- | --- | --- | --- | --- |
| **Parameter** | **Hazard-ratio (95% CI)** | **Median CNSM-FS (months)** | ***P*-value** | **Hazard-ratio**  **(95% CI)** | ***P*-value** |
| **SBR grade** |  |  | 0.0002 |  |  |
| I/II | 1 | 47.9 (40.2-64.2) |  |  |  |
| III | 1.28 (1.12-1.46) | 33.5 (28.6-39.1) |  |  |  |
| **Histological subtype** |  |  | 0.0089 |  |  |
| Ductal carcinoma | 1 | 35.4 (32.2-41.2) |  |  |  |
| Lobular carcinoma | 0.61 (0.44-0.85) | NR (NR-NR) |  |  |  |
| Other | 0.87 (0.62-1.22) | 40.9 (29.7-NR) |  |  |  |
| **Age at MBC diagnosis** |  |  | <0.0001 |  | **0.0006** |
| <50 | 1 | 35.1 (28.8-41.6) |  | 1 |  |
| [50-70] | 0.99 (0.87-1.13) | 33.6 (29.5-40.0) |  | 1.02 (0.88-1.18) |  |
| >70 | 0.61 (0.50-0.75) | NR (52.9-NR) |  | 0.68 (0.55-0.85) |  |
| **MFI (months)** |  |  | <0.0001 |  | **0.0005** |
| < 6 | 1 | 44.2 (37.3-52.9) |  | 1 |  |
| [6-24[ | 2.23 (1.88-2.64) | 16.9 (12.4-23.9) |  | 1.48 (1.16-1.88) |  |
| ≥ 24 | 1.24 (1.08-1.43) | 40.5 (34.0-51.7) |  | 1.04 (0.85-1.29) |  |
| **Number of metastatic sites at MBC diagnosis** |  |  | <0.0001 |  | **<0.0001** |
| <3 | 1 | 46.4 (40.9 (58.6) |  | 1 |  |
| ≥3 | 1.97 (1.72-2.27) | 15.6 (14.0-20.0) |  | 2.20 (1.90-2.55) |  |
| **ER status** |  |  | <0.0001 |  |  |
| Positive | 1 | 56.1 (46.3-69.2) |  |  |  |
| Negative | 1.75 (1.54-1.98) | 23.6 (21.2-28.0) |  |  |  |
| **PR status** |  |  | <0.0001 |  |  |
| Positive | 1 | 57.0 (47.5-NR) |  |  |  |
| Negative | 1.51 (1.32-1.73) | 31.0 (27.7-35.1) |  |  |  |
| **HR status** |  |  | <0.0001 |  | **<0.0001** |
| Positive | 1 | 54.1 (46.0-69.2) |  | 1 |  |
| Negative | 1.76 (1.55-1.99) | 23.3 (20.4-28.0) |  | 1.74 (1.52-1.99) |  |
| **Previous systemic treatment (*per os*)** |  |  | 0.6118 |  |  |
| No | 1 | 38.3 (33.8-43.3) |  |  |  |
| Yes | 1.09 (0.77-1.55) | 37.5 (24.9-NR) |  |  |  |
| **Previous systemic treatment (IV)** |  |  | <0.0001 |  |  |
| No | 1 | 48.4 (40.6-64.2) |  |  |  |
| Yes | 1.63 (1.44-1.85) | 29.1 (25.2-34.9) |  |  |  |
| **Previous HER2-targeted treatment** |  |  | <0.0001 |  | **<0.0001** |
| No |  | 49.9 (41.8-63.8) |  | 1 |  |
| Yes | 1.85 (1.64-2.10) | 23.7 (20.3-29.5) |  | 1.69 (1.39-2.04) |  |
| **Previous hormone therapy** |  |  | 0.1141 |  |  |
| No | 1 | 34.9 (30.8-40.6) |  |  |  |
| Yes | 0.90 (0.79-1.03) | 44.8 (38.0-65.3) |  |  |  |
| **Previous radiation therapy** |  |  | <0.0001 |  |  |
| No | 1 | 43.2 (37.3-50.6) |  |  |  |
| Yes | 1.41 (1.24-1.60) | 32.2 (27.7-40.5) |  |  |  |

CNS: Central Nervous System; CNSM: CNS Metastases; CNSM-FS: CNS Metastases-Free Survival; SBR grade: Scarff-Bloom-Richardson grade; MBC: Metastatic Breast Cancer; MFI: Metastases-Free Interval; NR: Not reached; ER: Estrogen Receptor; PR: Progesterone Receptor; HER2: Human Epidermal growth factor Receptor 2: IV: Intra Venous

**Supplementary Table 6.** Prognostic factors of OS in HER2-positive patients diagnosed with CNS metastases.

|  | **Univariate analysis** | | | **Multivariate analysis** | |
| --- | --- | --- | --- | --- | --- |
| **Parameter** | **Hazard-ratio (95% CI)** | **Median OS (months)** | ***P*-value** | **Hazard-ratio**  **(95% CI)** | ***P*-value** |
| **Age at CNSM diagnosis** |  |  | <0.0001 |  | **<0.0001** |
| <50 | 1 | 18.8 (16.0-21.6) |  | 1 |  |
| [50-70] | 1.19 (1.00-1.42) | 15.9 (13.4-18.8) |  | 1.13 (0.95-1.34) |  |
| >70 | 2.12 (1.66-2.72) | 5.7 (3.9-9.5) |  | 2.07 (1.62-2.66) |  |
| **Performance status at CNSM diagnosis^a^** |  |  | <0.0001 |  |  |
| 0 | 1 | 28.6 (24.8-65.1) |  |  |  |
| 1 | 1.31 (0.88-1.97) | 25.0 (20.0-NR) |  |  |  |
| 2 | 1.65 (1.00-2.72) | 21.7 (9.5-NR) |  |  |  |
| 3 | 5.10 (2.77-9.38) | 3.5 (2.9-NR) |  |  |  |
| 4 | 3.55 (0.85-14.79) | 10.5 (6.9-14.2) |  |  |  |
| **Time interval between breast cancer and CNSM diagnosis (months)** |  |  | 0.2034 |  |  |
| < 9 | 1 | 16.6 (14.1-19.4) |  |  |  |
| [9-18] | 1.08 (0.89-1.31) | 14.3 (12.9-19.3) |  |  |  |
| ≥ 18 | 1.19 (0.98-1.43) | 13.2 (10.7-18.8) |  |  |  |
| **ER status^b^** |  |  | 0.0005 |  |  |
| Positive | 1 | 19.9 (15.1-23.5) |  |  |  |
| Negative | 1.32 (1.13-1.54) | 13.1 (11.7-15.3) |  |  |  |
| **PR status^b^** |  |  | 0.0043 |  |  |
| Positive | 1 | 20.5 (14.9-25.2) |  |  |  |
| Negative | 1.29 (1.08-1.53) | 13.9 (12.2-16.1) |  |  |  |
| **HR status^b^** |  |  | 0.0002 |  | **<0.0001** |
| Positive | 1 | 19.9 (15.1-23.6) |  | 1 |  |
| Negative | 1.35 (1.15-1.57) | 12.8 (11.5-15.2) |  | 1.47 (1.26-1.72) |  |
| **Number of metastatic sites** |  |  | 0.0019 |  | **0.0094** |
| < 3 | 1 | 19.2 (16.1-21.8) |  | 1 |  |
| ≥ 3 | 1.28 (1.10-1.50) | 13.0 (11.4-15.0) |  | 1.24 (1.05-1.46) |  |
| **Number of previous chemotherapy lines** |  |  | <0.0001 |  | **<0.0001** |
| < 3 | 1 | 17.7 (15.9-20.0) |  | 1 |  |
| ≥ 3 | 2.08 (1.70-2.55) | 7.2 (5.9-8.8) |  | 2.09 (1.69-2.58) |  |
| **Previous HER2-targeted therapy** |  |  | <0.0001 |  | **0.0004** |
| No | 1 | 12.0 (9.8-13.5) |  | 1 |  |
| Yes | 0.73 (0.62-0.85) | 19.2 (16.6-21.5) |  | 0.75 (0.64-0.88) |  |

OS: Overall Survival; CNS: Central Nervous System; CNSM: CNS Metastases; CNSM-FS: CNS Metastases-Free Survival; ER: Estrogen Receptor; NR: Not reached; PR: Progesterone Receptor; HER2: Human Epidermal growth factor Receptor 2
^a^ Performance status was not included in the multivariate model due to a high number of missing data; ^b^ at MBC diagnosis, statuses defined as follows: status at the metastatic disease diagnosis, if available, or status of the primary tumor
